# Cystatin C and beta2-microglobulin: markers of glomerular filtration in critically ill children

**DOI:** 10.1186/cc5923

**Published:** 2007-05-22

**Authors:** José David Herrero-Morín, Serafín Málaga, Nuria Fernández, Corsino Rey, María Ángeles Diéguez, Gonzalo Solís, Andrés Concha, Alberto Medina

**Affiliations:** 1 Section of Paediatric Nephrology, Hospital Universitario Central de Asturias, Celestino Villamil Street, 33006, Oviedo, Spain and University of Oviedo, Julian Claveria Street, 33006, Oviedo, Spain; 2 Paediatrics Service, Hospital Cabueñes, Camino de los Prados Street, 395, 33204, Gijón, Spain; 3 Paediatric Intensive Care Unit, Department of Paediatrics, Hospital Universitario Central de Asturias, Celestino Villamil Street, 33006, Oviedo, Spain and University of Oviedo, Julian Claveria Street, 33006, Oviedo, Spain; 4 Immunology Unit, Department of Clinical Chemistry, Hospital Universitario Central de Asturias, Celestino Villamil Street, 33006, Oviedo, Spain and University of Oviedo, Julian Claveria Street, 33006, Oviedo, Spain

## Abstract

**Introduction:**

Parameters allowing regular evaluation of renal function in a paediatric intensive care unit (PICU) are not optimal. The aim of the present study was to analyse the utility of serum cystatin C and beta2-microglobulin (B2M) in detecting decreased glomerular filtration rate in critically ill children.

**Methods:**

This was a prospective, observational study set in an eight-bed PICU. Twenty-five children were included. The inverses of serum creatinine, cystatin C, and B2M were correlated with creatinine clearance (CrC) using a 24-hour urine sample and CrC estimation by Schwartz formula (Schwartz). The diagnostic value of serum creatinine, cystatin C, and B2M to identify a glomerular filtration rate under 80 ml/minute per 1.73 m^2 ^was evaluated using receiver operating characteristic (ROC) curve analysis.

**Results:**

Mean age was 2.9 years (range, 0.1 to 13.9 years). CrC was less than 80 ml/minute per 1.73 m^2 ^in 14 children, and Schwartz was less than 80 ml/minute per 1.73 m^2 ^in 9 children. Correlations between inverse of B2M and CrC (*r *= 0.477) and between inverse of B2M and Schwartz (*r *= 0.697) were better than correlations between inverse of cystatin C and CrC (*r *= 0.390) or Schwartz (*r *= 0.586) and better than correlations between inverse of creatinine and CrC (*r *= 0.104) or Schwartz (*r *= 0.442). The ability of serum cystatin C and B2M to identify a CrC rate and a Schwartz CrC rate under 80 ml/minute per 1.73 m^2 ^was better than that of creatinine (areas under the ROC curve: 0.851 and 0.792 for cystatin C, 0.802 and 0.799 for B2M, and 0.633 and 0.625 for creatinine).

**Conclusion:**

Serum cystatin C and B2M were confirmed as easy and useful markers, better than serum creatinine, to detect acute kidney injury in critically ill children.

## Introduction

Glomerular filtration rate (GFR) is difficult to measure in clinical practice [1-4]. The ideal laboratory marker should be of endogen synthesis, regular production rate, eliminated only by glomerular filtration, and without tubular secretion or reabsorption [4-6]. Creatinine clearance (CrC) using a 24-hour urine sample and serum creatinine (Cr) are the most commonly used parameters to estimate GFR in clinical practice [2,4,5,7,8], although not the most accurate. However, there are limitations to their use. Cr could be affected by factors other than renal function (for example, muscle mass, protein intake, inflammatory illness, or hepatic disease) [2,4,9-12]. Moreover, Cr is partially secreted by renal tubules [2,4,10,13] and frequently overestimates GFR [1,2,4,5,13]. On the other hand, CrC requires urine collection over a 24-hour period with a steady-state situation [1,2,4,11,14]. Mathematical formulas using Cr serum levels to estimate GFR (Schwartz formula is the most widely used and is based on Cr, age, and height) have been developed [15,16].

To overcome the problems of measuring GFR, an extensive search is being conducted to find a serum marker able to detect renal function impairment, especially at the initial phase. Cystatin C and beta2-microglobulin (B2M) are low-molecular-weight proteins freely filtered by the glomerulus [1,6,11,12]. Their serum concentrations, especially that of cystatin C, are less dependent on extra renal factors than in the case of Cr [1,5,6,10,11,13,14,17,18]. Early detection of renal function impairment in paediatric intensive care would be of great value, allowing accurate treatment, adjustment of drug dose, and prevention of more severe renal damage [3,7,9]. Previous studies demonstrated the superiority of serum cystatin C compared with creatinine in the evaluation of GFR [1,5,8,11,13,17-19], especially when there is a minor reduction in GFR [1,5,6,8,12,13]. We have not found any medical literature evaluating these low-molecular-weight proteins in critically ill children. The aim of this study was to evaluate the accuracies of serum Cr, serum cystatin C, and B2M as markers of GFR in critically ill children by comparing their results with CrC and Schwartz.

## Materials and methods

Twenty-five children admitted to our paediatric intensive care unit (PICU) were included in the study. All patients between the ages of 1 month and 14 years who were admitted due to an acute illness and who had a bladder catheter were included. The presence of previous renal or thyroideal pathology and the need of renal replacement therapy were considered exclusion criteria. Demographic and clinical conditions of the children were recorded. A serum sample was taken regularly from each patient in the morning (between 7 and 8 a.m.) for creatinine measurement. Cystatin C and B2M were measured in this sample. A 24-hour urine sample was obtained just before the serum sample to calculate the CrC adjusted to adult body surface area by means of the following formula: CrC (in millilitres/minute per 1.73 m^2^) = [(urine volume × urine Cr)/(serum Cr × 1,440)] × (1.73 m^2^/body surface). Schwartz CrC rate was calculated using the following formula: (height × k)/Cr, where height is calculated in centimetres, k = 0.44 for children under two years old and 0.55 for children over two years, and Cr is serum creatinine. Blood and urine samples were obtained 2.8 days (range, 1 to 6 days) after admission to the PICU. Serum and urine creatinine levels were measured using standard laboratory methods. Renal dysfunction was defined as CrC or as CrC estimation by Schwartz less than 80 ml/minute per 1.73 m^2^. Serum cystatin C and B2M levels were determined by endpoint nephelometry in a BN-II device (Dade Behring Marburg GmbH, Marburg, Germany).

Statistical analysis was performed using SPSS 11.0 (SPSS Inc., Chicago, IL, USA) and EPIDAT 3.0 (Xunta de Galicia, Galicia, Spain, and World Health Organization, 2003) for Windows. Data are expressed as a mean value and 95% confidence interval (CI) unless indicated otherwise. Inverses of Cr, cystatin C, and B2M were correlated with CrC and with Schwartz. We used the inverses of creatinine, cystatin C, and B2M to obtain a direct correlation with CrC and Schwartz formula. Correlations between age and CrC, Schwartz, creatinine, cystatin C, and B2M were performed. The diagnostic value of Cr, cystatin C, and B2M for identifying CrC or Schwartz less than 80 ml/minute per 1.73 m^2 ^was evaluated using receiver operating characteristic (ROC) curve analysis. Sensitivity, specificity, and positive likelihood ratio were calculated. A *p *value of less than 0.05 was considered statistically significant. Each patient's determinations were made with the informed consent of their parents.

## Results

The mean age was 2.9 years (95% CI, 1.4 to 4.3 years) with a range of 0.1 to 13.9 years and a median of 1.3 years. Male/female ratio was 1.27:1. Mean height was 86.3 cm (range, 53.0 to 151.0 cm). Mean body surface area was 0.5 m^2 ^(range, 0.2 to 1.5 m^2^). Mean paediatric risk of mortality (PRISM) [20] (standard deviation [SD]) scores 24 hours after admission were 15.0 (11.3). The patients' clinical conditions and the treatments they received are summarized in Table 1.

**Table 1:** Clinical conditions and treatments patients received during urine collection

Parameter	Number (percentage) of patients
Disease	
Infectious disease	7 (28)
Multiple trauma	4 (16)
Respiratory disease	4 (16)
Metabolic disease	4 (16)
Postsurgical high risk	3 (12)
Neurological disease	3 (12)

Treatment	
Mechanical ventilation	13 (52)
Inotropic drugs	12 (48)
Diuretics (bolus)	9 (36)
Diuretics (continuous perfusion)	3 (12)
Enteral nutrition	14 (56)

The mean CrC was 76.3 ml/minute per 1.73 m^2 ^(95% CI, 58.4 to 94.1 ml/minute per 1.73 m^2^) and the mean Schwartz was 104.5 ml/minute per 1.73 m^2 ^(95% CI, 88.3 to 120.8 ml/minute per 1.73 m^2^). The mean serum Cr concentration was 0.42 mg/dl (95% CI, 0.36 to 0.48 mg/dl), the mean serum cystatin C concentration was 0.69 mg/l (95% CI, 0.57 to 0.81 mg/l), and the mean serum B2M concentration was 2.12 mg/l (95% CI, 1.66 to 2.57 mg/l). There were no significant differences between male and female regarding CrC (79.9 versus 71.7 ml/minute per 1.73 m^2^), Schwartz (103.7 versus 105.5 ml/minute per 1.73 m^2^), serum Cr (0.43 versus 0.42 mg/dl), serum cystatin C (0.65 versus 0.75 mg/l), and serum B2M (2.23 versus 1.97 mg/l).

Fourteen out of the 25 patients enrolled in the study (56.0%) had a CrC less than 80 ml/minute per 1.73 m^2^, and 9 patients (36%) had a Schwartz less than 80 ml/minute per 1.73 m^2^. CrC, Schwartz, serum Cr, serum cystatin C, and serum B2M values are listed in Table 2, separating patients in two groups: renal dysfunction (CrC less than 80 ml/minute per 1.73 m^2^) and normal renal function. Schwartz, CrC, serum Cr, serum cystatin C, and serum B2M are listed in Table 3, separating patients in two groups: renal dysfunction (Schwartz less than 80 ml/minute per 1.73 m^2^) and normal renal function.

**Table 2:** Comparison of groups with creatinine clearance lower and higher than 80 ml/minute per 1.73 m^2^

	CrC < 80 ml/minute per 1.73 m^2^		CrC > 80 ml/minute per 1.73 m^2^		*P *value
	
	N	Mean (95% CI)	N	Mean (95% CI)	
CrC	14	47.7 (40.6–54.8)	11	112.6 (85.0–140.3)	< 0.01
Schwartz	14	82.8 (64.6–100.9)	11	132.2 (111.7–152.7)	< 0.01
Serum Cr (mg/dl)	14	0.45 (0.36–0.54)	11	0.38 (0.29–0.47)	NS
Serum cystatin C (mg/l)	14	0.83 (0.67–0.99)	11	0.51 (0.38–0.64)	< 0.01
Serum B2M (mg/l)	14	2.60 (1.94–3.26)	11	1.50 (1.02–1.90)	< 0.05

Creatinine clearance (CrC), creatinine clearance estimation by Schwartz formula (Schwartz), serum creatinine (Cr), serum cystatin C, and serum beta2-microglobulin (B2M) in patients with renal dysfunction (CrC less than 80 ml/minute per 1.73 m^2^) and normal renal function are shown. CI, confidence interval; N, number of patients; NS, not significant.

**Table 3:** Comparison of groups with creatinine clearance estimation by Schwartz formula lower and higher than 80 ml/minute per 1.73 m^2^

	Schwartz < 80 ml/minute per 1.73 m^2^		Schwartz > 80 ml/minute per 1.73 m^2^		*P *value
	
	N	Mean (95% CI)	N	Mean (95% CI)	
Schwartz	9	68.8 (63.2–74.3)	16	124.6 (105.8–143.5)	< 0.01
CrC	9	54.5 (48.4–60.6)	16	88.5 (61.8–115.2)	< 0.05
Serum Cr (mg/dl)	9	0.45 (0.35–0.56)	16	0.41 (0.32–0.49)	NS
Serum cystatin C (mg/l)	9	0.82 (0.67–0.97)	16	0.62 (0.45–0.79)	NS
Serum B2M (mg/l)	9	2.73 (1.94–3.52)	16	1.77 (1.23–2.33)	< 0.05

Creatinine clearance estimation by Schwartz formula (Schwartz), creatinine clearance (CrC), serum creatinine (Cr), serum cystatin C, and serum beta2-microglobulin (B2M) in patients with renal dysfunction (Schwartz less than 80 ml/minute per 1.73 m^2^) and normal renal function are shown. CI, confidence interval; N, number of patients; NS, not significant.

Mean PRISM (SD) scores were 13.5 (14.5) in the group with CrC less than 80 ml/minute per 1.73 m^2 ^and 10.2 (5.1) in the group with CrC greater than 80 ml/minute per 1.73 m^2 ^(differences not significant [NS]). Mean PRISM (SD) scores were 15.9 (16.5) in the group with Schwartz less than 80 ml/minute per 1.73 m^2 ^and 9.6 (5.7) in the group with Schwartz greater than 80 ml/minute per 1.73 m^2 ^(differences NS).

The correlation with CrC was better for the inverse of serum B2M (*r *= 0.477, *p *< 0.05) than for the inverse of cystatin C (*r *= 0.390, *p *= 0.054). The inverse of Cr showed the worse correlation with CrC (*r *= 0.104, differences NS). The correlation with Schwartz was also better for the inverse of serum B2M (*r *= 0.697, *p *< 0.01) than for the inverse of cystatin C (*r *= 0.586, *p *< 0.01). The inverse of Cr showed the worse correlation with Schwartz (*r *= 0.442, *p *< 0.05).

Patient age in the renal dysfunction group was significantly lower than in the normal renal function group, 1.6 years (95% CI, 0.3 to 2.8 years) versus 4.7 years (95% CI, 1.8 to 7.5 years) if GFR was estimated with CrC and 0.9 years (95% CI, 0.3 to 1.5 years) versus 4.1 years (95% CI, 1.9 to 6.2 years) if GFR was estimated with Schwartz. Age had a lineal correlation with CrC (*r *= 0.42, *p *< 0.05), Schwartz (*r *= 0.41, *p *< 0.05), and Cr (*r *= 0.54, *p *< 0.01), but not with cystatin C (*r *= 0.10, differences NS) and B2M (*r *= 0.23, differences NS).

**Figure 1:**
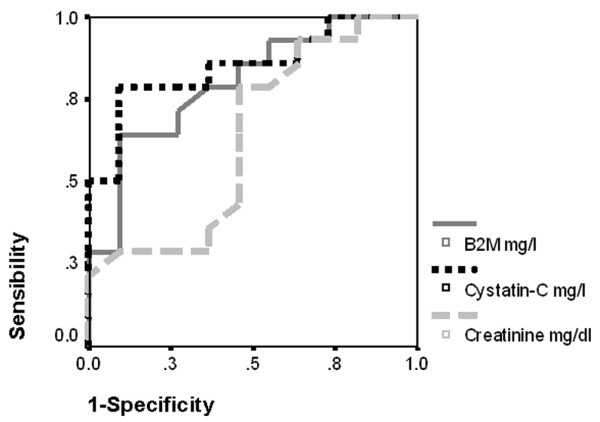
Receiver operating characteristic curves to diagnose creatinine clearance of less than 80 ml/minute per 1.73 m^2^. Areas under the curve (95% confidence intervals) are 0.633 (0.403 to 0.863) for creatinine, 0.851 (0.698 to 1.003) for cystatin C, and 0.802 (0.628 to 0.976) for beta2-microglobulin (B2M).

**Figure 2:**
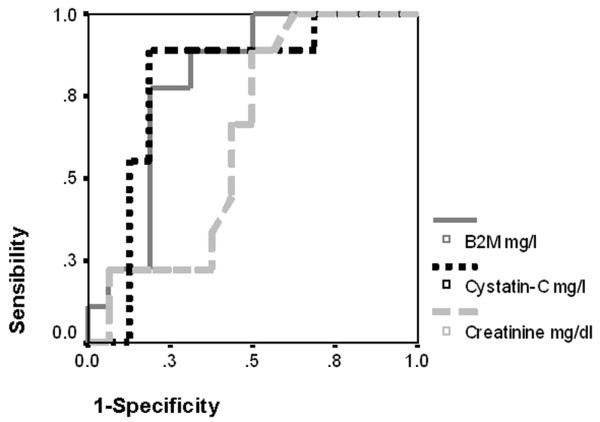
Receiver operating characteristic curves to diagnose creatinine clearance estimation by Schwartz formula of less than 80 ml/minute per 1.73 m^2^. Areas under the curve (95% confidence intervals) are 0.625 (0.403 to 0.847) for creatinine, 0.792 (0.598 to 0.986) for cystatin C, and 0.799 (0.622 to 0.976) for beta2-microglobulin (B2M).

ROC curves for detecting renal dysfunction are shown in Figures 1 and 2. To diagnose CrC less than 80 ml/minute per 1.73 m^2^, the areas under the curve were 0.633 (95% CI, 0.403 to 0.863) for serum Cr, 0.851 (95% CI, 0.698 to 1.003) for serum cystatin C, and 0.802 (95% CI, 0.628 to 0.976) for serum B2M. To diagnose Schwartz less than 80 ml/minute per 1.73 m^2^, the areas under the curve were 0.625 (95% CI, 0.403 to 0.847) for serum Cr, 0.792 (95% CI, 0.598 to 0.986) for serum cystatin C, and 0.799 (95% CI, 0.622 to 0.976) for serum B2M.

The diagnosis efficacy of each marker we studied to detect renal dysfunction is shown in Tables 4 and 5. For each variable, the cutoff level with best sensitivity and specificity was chosen.

**Table 4:** Diagnostic efficiency values for serum creatinine, cystatin C, and beta2-microglobulin to detect early renal dysfunction

	Serum creatinine	Cystatin C	Beta2-microglobulin
Cutoff value	0.4 mg/dl	0.6 mg/l	1.5 mg/l
Sensitivity	42% (39%–46%)	85% (82%–89%)	85% (82%–89%)
Specificity	54% (49%–59%)	63% (58%–68%)	54% (49%–59%)
Positive likelihood ratio	0.94 (0.94–0.95)	2.3 (2.3–2.3)	1.8 (1.8–1.8)

Sensitivity, specificity, and positive likelihood ratio (95% confidence interval) for serum creatinine, cystatin C, and beta2-microglobulin to diagnose renal dysfunction (creatinine clearance less than 80 ml/minute per 1.73 m^2^) are shown.

**Table 5:** Diagnostic efficiency values for serum creatinine, cystatin C, and beta2-microglobulin to detect early renal disfunction by Schwartz formula

	Serum creatinine	Cystatin C	Beta2-microglobulin
Cutoff value	0.4 mg/dl	0.6 mg/l	1.5 mg/l
Sensitivity	44% (38%–50%)	88% (83%–94%)	100% (94%–100%)
Specificity	56% (52%–59%)	50% (46%–53%)	50% (46%–53%)
Positive likelihood ratio	1.0 (1.0–1.0)	1.7 (1.7–1.8)	2.0 (1.9–2.0)

Sensitivity, specificity, and positive likelihood ratio (95% confidence interval) for serum creatinine, cystatin C, and beta2-microglobulin to diagnose renal dysfunction (creatinine clearance estimation by Schwartz formula less than 80 ml/minute per 1.73 m^2^) are shown.

## Discussion

Gold standard techniques to assess GFR are based on exogenous substance clearance (inulin, iodine 125-iothalamate, technetium 99m-DTPA [diethylenetriaminepentaacetic acid], chromium 51-EDTA [ethylenediaminetetraacetic acid], ...) [1-4,21]. However, these methods are difficult to apply in clinical practice [1,3,7,10,13,14,21,22]. We chose CrC and Schwartz formula as gold standards because they are commonly used in clinical practice. Because urine collection technique during 24 hours is a limiting factor in children, we included only those children who had a bladder catheter in place. Serum Cr level is frequently used in daily practice [2,4,5,7,8]. However, only two criteria of an ideal GFR marker are accomplished by serum Cr: endogenous substance and free glomerular filtration [11]. Serum Cr level is affected by circumstances other than renal ones [2,4,9-12,21] and it is secreted by the renal tubules [2,4,10,13]. This leads to an overestimation of GFR, especially when a moderate GFR descent is present [1,2,4,5,13,21]. Serum Cr could not detect renal failure until GFR decreases more than 50% [10,12]. Our results showed that serum Cr levels were not statistically different in the groups with a level higher or lower than 80 ml/minute per 1.73 m^2^, confirming the low sensitivity of serum Cr to detect renal dysfunction. In critically ill children, there would be a muscle loss and a relative malnutrition; in these cases, serum Cr could also indicate GFR values higher than the actual levels [2,10,12,23].

Cystatin C and B2M are low-molecular-weight proteins produced by all nucleated cells at a constant rate [1,2,4-6,8,9,11-14,18,24]. They are freely filtered by the glomerulus and reabsorbed and catabolized by proximal tubular cells [2,4-6,8,9,11-14,18,23,24]. Therefore, their serum values could be a better marker of GFR than serum Cr level [1,6,24].

The molecular weight of cystatin C is 13.3 kDa [1,2,4,5,8,9,11,14,18,22,24]. Its concentration is more dependent on renal function in comparison with serum Cr [1,14,21,23]. However, some authors found that serum cystatin C concentration could increase in hyperthyroidism or in patients who are receiving corticosteroids [3,8,11,18,21,23], although other studies did not find this association [1,15,25].

The molecular weight of B2M is 11.8 kDa [6,22]. It is not influenced by age [5,17], gender, or muscle mass [12,19]. However, unlike production of cystatin C, that of B2M increases in infectious or inflammatory process, proliferative syndromes, and hepatic and autoimmune illnesses [1,5,6,12,26].

Several studies have demonstrated the capacity of cystatin C to estimate GFR [8,9,11,13,14,18,19,24] and that its ability to detect moderate acute kidney injury was better than that of Cr [1,5,6,8,13,19,23,24]. Cystatin C values will be abnormally high when GFR decreases to 88 to 95 ml/minute per 1.73 m^2 ^[6,13,19]. Therefore, cystatin C could detect renal dysfunction one to two days before Cr [8]. Cystatin C was also superior to Cr in children and patients with muscle loss [5,14,18]. Recent studies have developed formulas that include cystatin C [15,16]. Some of them also include serum Cr and other factors like age, weight, and height, improving diagnostic accuracy. Other authors have shown that parameters such as urinary neutrophil gelatinase-associated lipocalin and interleukin-18 were superior to serum Cr for acute kidney injury diagnosis [27-29]. However, new evaluations will be needed. B2M also increases before Cr [12,19]. Some studies found B2M to be less adequate than cystatin C as a GFR marker [18,24,26], but one other did not show any differences [6].

It has been demonstrated that low-molecular-weight proteins have greater diagnostic sensitivity than serum Cr in children [17,25] and critically ill adults [7,9,23]. However, to date, no studies have been performed in critically ill children.

We found CrC less than 80 ml/minute per 1.73 m^2 ^in 56% and Schwartz less than 80 ml/minute per 1.73 m^2 ^in 37.5% of the cases, similar to other studies with critically ill patients [7,9]. Patients with CrC less than 80 ml/minute per 1.73 m^2 ^and with Schwartz less than 80 ml/minute per 1.73 m^2 ^were younger than patients with normal renal function. Like previous studies [1,12,17-19,25], we did not find gender differences in cystatin C and B2M levels.

As shown in Figures 1 and 2, we found that the capacities of cystatin C and B2M to detect acute kidney injury were better than that of serum Cr. Filler and colleagues [17] obtained similar results to detect patients with GFR less than 90 ml/minute per 1.73 m^2 ^in 225 children with different chronic renal diseases; the areas under the ROC curve were 0.840, 0.943, and 0.899 for serum Cr, cystatin C, and B2M, respectively. Our study showed worse figures for serum Cr, with areas under the ROC curve of 0.633 and 0.625. The probable explanation is that our patients were critically ill. In these patients, serum Cr demonstrated an insufficient sensibility to detect early acute kidney injury. Hoste and colleagues [7] and Delanaye and colleagues [23] found normal levels of serum Cr in critically ill adults when 46.4% and 42% of them, respectively, had CrC less than 80 ml/minute per 1.73 m^2^. Villa and colleagues [9] and Delanaye and colleagues [23] found a better correlation of CrC with the inverse of cystatin C than with the inverse of Cr (*r *= 0.832 versus 0.426 and *r *= 0.68 versus 0.4, respectively). They also found a higher cystatin C sensitivity to detect CrC less than 80 ml/minute per 1.73 m^2 ^(areas under the ROC curve, 0.927 versus 0.694 and 0.833 versus 0.789, respectively). Le Bricon and colleagues [14] found the same results in critically ill adults.

In critically ill children, early diagnosis of renal impairment is very important for making therapeutic decisions [3,7,9]. Therefore, we tried to determine the best marker to detect CrC and Schwartz less than 80 ml/minute 1.73 m^2^. Cystatin C and B2M had a better area under the ROC curve than Cr. Cystatin C and B2M had better diagnostic efficiency values than Cr (Tables 4 and 5). As previously shown in other groups of patients [5], in our experience serum cystatin C and B2M are better markers than serum Cr to detect acute kidney injury in critically ill children. One possible explanation is that GFR in critically ill children can change rapidly, but changes in serum Cr take more time.

We obtained cutoff values to differentiate patients with CrC and with Schwartz less than 80 ml/minute 1.73 m^2 ^that were considered normal in previous studies [5,11,17,18,30]. A possible explanation was the early determination of the biological markers at the beginning of renal dysfunction. Longitudinal assessment of GFR markers over a period of time will be useful to determine their value for the early diagnosis of GFR decrease.

Patients with CrC and Schwartz less than 80 ml/minute 1.73 m^2 ^were younger. Age correlates with Cr but not with cystatin C and B2M. Therefore, age did not influence the higher cystatin C concentration found in patients with low CrC or low Schwartz.

Our study has limitations. The main problem is the 'gold standard' against which low-molecular-weight proteins can be calibrated. CrC using a 24-hour urine sample and Schwartz formula based on serum Cr and height were considered the gold standard because, despite the controversy, they are used in clinical practice. Another limitation of our study was the size of the sample and the difference in the ages of the patients. Because of the small size of the sample, we cannot compare children older than one year and those younger than one year.

## Conclusion

In our experience, serum cystatin C and B2M were confirmed as simple and useful markers, better than serum Cr, to detect acute kidney injury in critically ill children. However, new studies with a bigger sample of patients and more accurate gold standards to use for comparisons will be necessary to establish cystatin C or B2M as biochemical markers for monitoring GFR in unstable critically ill children.

## Key messages

• Glomerular filtration rate is difficult to measure in clinical practice, and the most commonly used parameters (serum creatinine and creatinine clearance) are not the most accurate.

• Cystatin C and beta2-microglobulin are low-molecular-weight proteins freely filtered by the glomerulus, the serum concentration of which is less dependent on extra renal factors than serum creatinine.

• In our study, correlations with creatinine clearance and creatinine clearance estimated by Schwartz formula are better for cystatin C and beta2-microglobulin than for serum creatinine.

• The area under the receiver operating characteristic curves of cystatin C and beta2-microglobulin to diagnose creatinine clearance and creatinine clearance estimated by Schwartz formula less than 80 ml/minute per 1.73 m^2 ^are greater than the one for serum creatinine in our study.

• In our experience, serum cystatin C and beta2-microglobulin are confirmed as simple and useful markers, better than serum creatinine, to detect acute kidney disease in critically ill children.

## Abbreviations

B2M = beta2-microglobulin; CI = confidence interval; Cr = serum creatinine; CrC = creatinine clearance; GFR = glomerular filtration rate; NS = not significant; PICU = paediatric intensive care unit; PRISM = paediatric risk of mortality; ROC = receiver operating characteristic; Schwartz = creatinine clearance estimation by Schwartz formula; SD = standard deviation.

## Competing interests

This study was supported in part by an Ernesto Sánchez Villares Foundation fellowship. This foundation belongs to the SCCALP (a regional section of the Spanish Paediatric Association). The authors declare that they have no other competing interests.

## Authors' contributions

JDH-M participated in the design of the study, revision of the medical literature, data collection, and statistics analysis and drafted the manuscript. SM participated in the design of the study and helped to draft the manuscript. NF participated in the medical literature revision and helped to draft the manuscript. CR, AC, and AM participated in the data collection and helped to draft the manuscript. MAD carried out the laboratory determinations and participated in the interpretation of the laboratory results. GS carried out the statistical analysis and helped to draft the manuscript. All authors read and approved the final manuscript.

## References

[B1] Kezama JJ, Kutsuwada K, Ataka K, Maruyama H, Gejyo F (2002). Serum cystatin C reliably detects renal dysfunction in patients with various renal diseases. Nephron.

[B2] Rosner MH, Bolton WK (2006). Renal function testing. Am J Kidney Dis.

[B3] Grubb A, Nyman U, Björk J, Lindström V, Rippe B, Sterner G, Christensson A (2005). Simple cystatin C-based prediction equations for glomerular filtration rate compared with the modification of diet in renal disease prediction equation for adults and the Schwartz and the Counahan-Barratt prediction equations for children. Clin Chem.

[B4] Stevens LA, Levey AS (2005). Measurement of kidney function. Med Clin N Am.

[B5] Laterza OF, Price CP, Scott MG (2002). Cystatin C: an improved estimator of glomerular filtration rate?. Clin Chem.

[B6] Jovanovic D, Krstivojevic P, Obradovic I, Durdevic V, Dukanovic L (2003). Serum cystatin C and β2-microglobulin as markers of glomerular filtration rate. Ren Fail.

[B7] Hoste EA, Damen J, Vanholder RC, Lameire NH, Delanghe JR, Van der Hauwe K, Colardyn FA (2005). Assessment of renal function in recently admitted critically ill patients with normal serum creatinine. Nephrol Dial Transplant.

[B8] Herget-Rosenthal S, Marggraf G, Hüsing J, Göring F, Pietruck F, Janssen O, Philipp T, Kribben A (2004). Early detection of acute renal failure by serum cystatin C. Kidney Int.

[B9] Villa P, Jiménez M, Soriano MC, Manzanares J, Casasnovas P (2005). Serum cystatin C concentration as a marker of acute renal dysfunction in critically ill patients. Crit Care.

[B10] Huber AR, Risch L (2005). Recent developments in the evaluation of glomerular filtration rate: is there a place for beta-trace?. Clin Chem.

[B11] Newman DJ (2002). Cystatin C. Ann Clin Biochem.

[B12] Bianchi C, Donadio C, Tramonti G, Consani C, Lorusso P, Rossi G (2001). Reappraisal of serum β2-microglobulin as marker of GFR. Ren Fail.

[B13] Coll E, Botey A, Alvarez L, Poch E, Quintó L, Taurina A, Vera M, Piera C, Darnell A (2000). Serum cystatin C as a new marker for noninvasive estimation of glomerular filtration rate and as a marker for early renal impairment. Am J Kidney Dis.

[B14] Le Bricon TL, Leblanc I, Benlakehal M, Gay-Bellile C, Erlich D, Boudaoud S (2005). Evaluation of renal function in intensive care: plasma cystatin C vs creatinine and derived glomerular filtration rate estimates. Clin Chem Lab Med.

[B15] Bouvet Y, Bouissou F, Coulais Y, Séronie-Vivien S, Tafani M, Decramer S, Chatelut E (2006). GFR is better estimated by considering both serum cystatin C and creatinine levels. Pediatr Nephrol.

[B16] Zappitelli M, Parvex P, Joseph L, Paradis G, Grey V, Lau S, Bell L (2006). Derivation and validation of cystatin C-based predition equations for GFR in children. Am J Kidney Dis.

[B17] Filler G, Priem F, Lepage N, Sinha P, Vollmer I, Clark H, Keely E, Matzinger M, Akbari A, Althaus H (2002). β-Trace protein, cystatin C, β_2_-microglobulin, and creatinine compared for detecting impaired glomerular filtration rates in children. Clin Chem.

[B18] Filler G, Bökenkamp A, Hofmannn W, Le Bricon T, Martínez-Brú C, Grubb A (2005). Cystatin C as a marker of GFR-history, indications, and future research. Clin Biochem.

[B19] John GT, Fleming JJ, Talaulikar GS, Servakumar R, Thomas PP, Jacob CK (2003). Measurement of renal function in kidney donors using serum cystatin C and β_2_-microglobulin. Ann Clin Biochem.

[B20] Pollack MM, Ruttimann UE, Getson PR (1988). Pediatric risk of mortality (PRISM) score. Crit Care Med.

[B21] Stevens LA, Coresh J, Greene T, Levey AS (2006). Assessing kidney function-measured and estimated glomerular filtration rate. N Engl J Med.

[B22] Donadio C, Lucchesi A, Ardini M, Giordani R (2001). Cystatin C, β2-microglobulin, and retinol-binding protein as indicators of glomerular filtration rate: comparison with plasma creatinina. J Pharm Biomed Anal.

[B23] Delanaye P, Lambermont B, Chapelle JP, Gielen J, Gerard P, Rorive G (2004). Plasmatic cystatin C for the estimation of glomerular filtration rate in intensive care units. Intensive Care Med.

[B24] Grubb AO (2000). Cystatin C – properties and use as diagnostic marker. Adv Clin Chem.

[B25] Foster J, Reisman W, Lepage N, Filler G (2006). Influence of commonly used drugs on the accuracy of cystatin C-derived glomerular filtration rate. Pediatr Nephrol.

[B26] Grubb A, Simonsen O, Sturfelt G, Truedsson L, Thysell H (1985). Serum concentration of cystatin C, factor D and β2-microglobulin as a measure of glomerular filtration rate. Acta Med Scand.

[B27] Parikh CR, Jani A, Melnikov VY, Faubel S, Edelstein CL (2004). Urinary interleukin-18 is a marker of human acute tubular necrosis. Am J Kidney Dis.

[B28] Parikh CR, Jani A, Mishra J, Ma Q, Kelly C, Barasch J, Edelstein CL, Devarajan P (2006). Urine NGAL and IL-18 are predictive biomarkers for delayed graft function following kidney transplantation. Am J Transplant.

[B29] Parikh CR, Mishra J, Thiesssen-Philbrook H, Dursun B, Ma Q, Kelly C, Dent C, Devarajan P, Edelstein CL (2006). Urinary IL-18 is an early predictive biomarker of acute kidney injury after cardiac surgery. Kidney Int.

[B30] Randers E, Krue S, Erlandsen EJ, Danielsen H, Hansen LG (1999). Reference interval for serum cystatin C in children. Clin Chem.

